# Population Health Management for Improving Kidney Health Outcomes

**DOI:** 10.1053/j.ajkd.2025.01.020

**Published:** 2025-03-17

**Authors:** Manisha Jhamb, Jane O. Schell, Melanie R. Weltman, Linda-Marie U. Lavenburg, Chethan Puttarajappa, Gary S. Fischer, Thomas Kleyman

**Affiliations:** Renal-Electrolyte Division (MJ, JOS, MRW, L-MUL, CP, TK) and Division of General Internal Medicine (JOS, GSF), Department of Pharmacy and Therapeutics, School of Pharmacy (MRW), University of Pittsburgh, Pittsburgh, Pennsylvania

## Abstract

Chronic kidney disease (CKD) is globally prevalent, a leading cause of mortality, and is associated with poor patient outcomes and high health care costs. Gaps in guideline-concordant care are common across the continuum of CKD. These gaps lead to CKD progression, hospitalizations, and mortality and are potentiated by existing racial and socioeconomic disparities. A thoughtfully designed population health management approach that leverages electronic health records can modernize CKD care delivery and improve outcomes. Such an approach can potentially provide timely, equitable, resource-and cost-efficient care across health systems in a way that is scalable and data driven. Herein, we share our experiences with the implementation of nephrology population health initiatives at the University of Pittsburgh Medical Center across the CKD spectrum, which include ongoing and planned programs in the primary care, kidney-palliative care, kidney transplantation, and transitions of care settings. Further, we discuss the challenges of population health management and future directions that can move health care toward personalized medicine.

The rising global burden of chronic kidney disease (CKD) has prompted an urgent call for action at the health system, payor, policy, clinician, and researcher levels to foster innovation for “closing the gap between what we know and what we do” for improving CKD care.^1,2^ In the United States, CKD affects an estimated 33.5 million people, about 15% of the adult population.^3^ Kidney disease is the tenth leading cause of death globally and is associated with negative consequences, including disability, reduced quality of life, psychosocial harm, and premature mortality.^3,4^ Kidney disease–related Medicare expenditures are approximately $137 billion, of which over a third is spent on patients with end-stage kidney disease (ESKD), who represent <5% of patients with CKD.^5^ Recently, international organizations have called for inclusion of CKD in the World Health Organization’s noncommunicable disease category to enhance its global awareness and need for urgent action.^4^

Gaps in CKD care include poor patient education and awareness,^3,6^ inadequate diagnostic evaluation,^7,8^ medication errors,^9,10^ suboptimal management,^2,11,12^ and delayed nephrology referral.^13,14^ These shortcomings inevitably lead to CKD progression, hospitalizations, and mortality.^13–15^ Unfortunately, these gaps in guideline-concordant CKD care have persisted over the last decade^2,16,17^ and may widen further with rapidly evolving guidelines, increasing patient complexity, and care fragmentation.^18^ Early data on newer kidney-protective and cardioprotective medications show that in 2021 only 9%-11% of Medicare beneficiaries with CKD (stages 3-5) and type 2 diabetes were prescribed glucagon-like peptide 1 (GLP1) receptor agonists or sodium/glucose cotransporter 2 (SGLT2) inhibitors.^19,20^ Moreover, racial and ethnic disparities exist in CKD care, transplant referral, home dialysis uptake, and access to nephrology specialists and newer medications.^16,21,22^ Finally, current CKD care is associated with substantial care fragmentation, duplication of testing, and patient inconvenience and travel burden, affecting a predominantly older, comorbid population.

## The Promise of Population Health Management in Kidney Disease

The 2019 Advancing American Kidney Health initiative calls for public health strategies and implementation research to improve early detection and evidence-based CKD care to reduce ESKD incidence in the United States by 25% by 2030.^23^ Achieving this ambitious target requires focus on value-based care (VBC) and perhaps alternative payment models to improve care quality. VBC programs in nephrology, such as the ESKD Seamless Care Organizations (ESCOs) and Kidney Care Choices (KCC), have largely focused on late-stage CKD and ESKD.^24,25^ A shift toward early-stage CKD is needed to delay progression and reduce complications, including cardiovascular morbidity and mortality. A population health management (PHM) strategy may provide such an opportunity to improve the continuum of kidney health and optimize quality, safety, and the cost of managing kidney disease. Such an approach may deliver value-based CKD care that is high-impact, resource efficient, scalable, and equitable.

**Figure 1.**
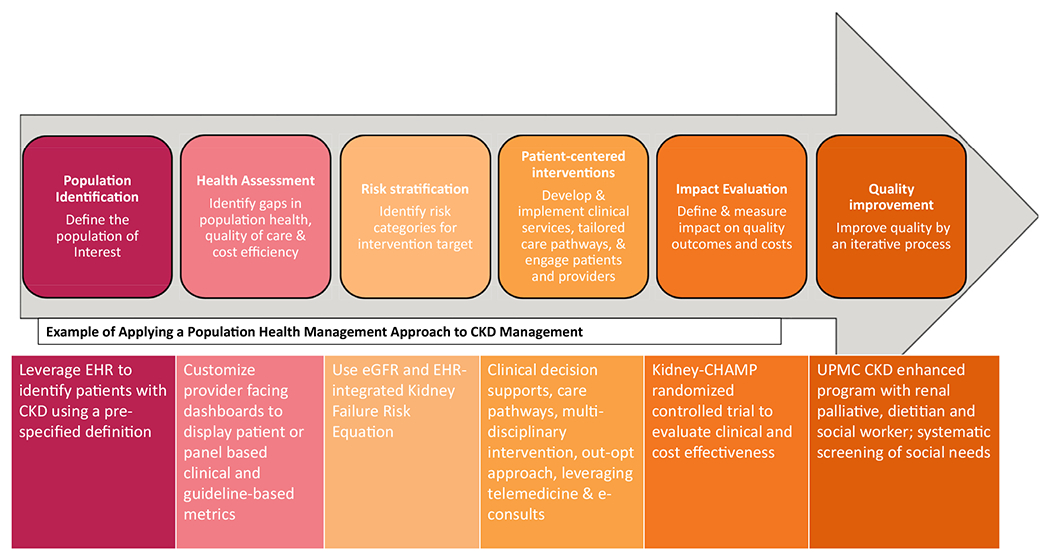
Population health management model and its application to chronic kidney disease management. Abbreviations: CHAMP, Coordinated Health Management Partnership; CKD, chronic kidney disease; eGFR, estimated glomerular filtration rate; EHR, electronic health record; UPMC, University of Pittsburgh Medical Center.

PHM is described as a “proactive, organized, and cost-effective approach to prevention that utilizes newer technologies to reduce morbidity while improving the health status, health service use, and personal productivity of individuals in defined populations.”^26^ It includes both prevention and chronic management and uses aggregate population data to risk stratify and individualize care pathways to maximize resource-efficiency and quality of care.^27^ The Care Continuum Alliance PHM model^28^ and its analytical framework describe the core elements as (1) population identification, (2) health assessment, (3) risk stratification, (4) implementation of patient-centered interventions, (5) impact evaluation, and (6) iterative quality improvement (Fig 1).

**Figure 2.**
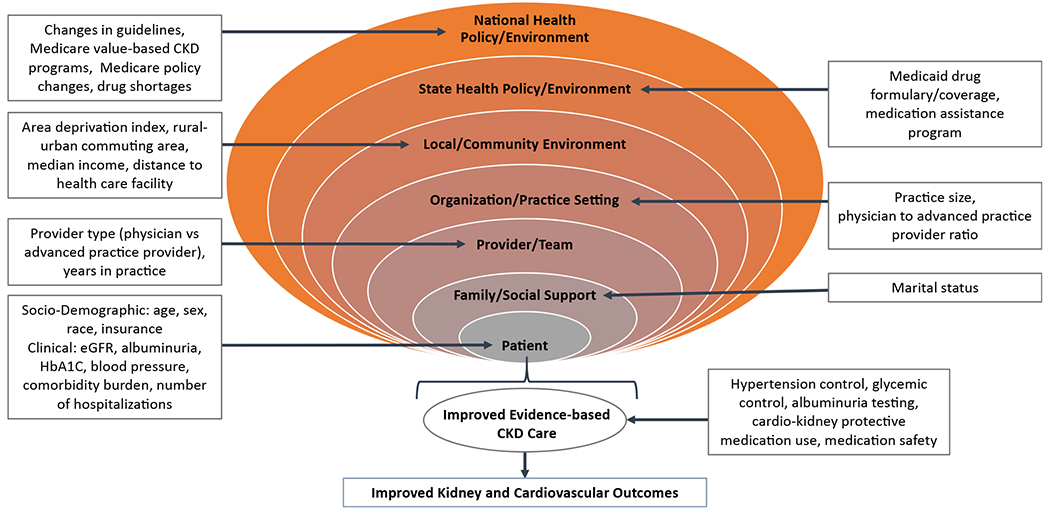
Contextual factors at micro, meso, and macro levels that can affect PHM for kidney disease. Accounting for factors at patient, provider, community, health system, and policy levels is essential to ensure the PHM approach is feasible, acceptable, and aligns with overall goal of improving population kidney health. Abbreviations: CKD, chronic kidney disease; eGFR, estimated glomerular filtration rate; HbA1c, hemoglobin A_1c_; PHM, population health management.

A PHM strategy for CKD that leverages electronic health records (EHRs) and a multidisciplinary team approach can address barriers at the patient, provider, and health system levels. PHM can accelerate implementation and adoption of standardized treatments and guideline-based care pathways to narrow gaps in CKD care while also improving access to racial and ethnic minorities and rural populations to promote health equity. Designing a successful PHM approach for CKD requires thoughtful consideration of the myriad contextual factors at micro, meso, and macro levels that affect kidney health and care delivery in an ecosystem (Fig 2).

This perspective will describe our experiences with implementing nephrology-based population health initiatives at the University of Pittsburgh Medical Center (UPMC). UPMC is one of the few fully integrated health systems in the United States with its own insurance plan, which covers more than 4 million members, so it provides an ideal environment for innovative models of care. UPMC’s not-for-profit health care system has a network of over 40 tertiary care, specialty, and community hospitals and 800 doctors’ offices and outpatient sites in Pennsylvania, New York, and Maryland. The diverse patient population, heterogenous provider settings, and mature EHR with a strong clinical informatics infrastructure has allowed us to develop and test the PHM strategies.

## Population Health Management Across Kidney Disease Spectrum

A PHM program across the kidney disease continuum, including kidney transplant and ESKD, can provide a comprehensive strategy for addressing gaps in care and improving patient outcomes. Because several prior and ongoing initiatives for VBC in ESKD have been well-described,^24,29,30^ we now describe our vision and challenges for PHM across nondialysis CKD stages. We also share a “toolbox” with critical elements that are required to establish a CKD PHM program in any health system (Box 1).

## PHM Implementation Strategy for CKD

### Building a Strong PHM Infrastructure to Identify Care Gaps

To operationalize PHM for CKD, a strong informatics infrastructure and a data-driven approach are critical. To this end, we developed a CKD registry within the EHR (EpicCare) that includes all outpatients with a CKD diagnosis and/or eGFR < 60 mL/min/1.73 m^2^ (based on the 2 most recent outpatient eGFR measures at least 90 days apart). The registry was based on similar initiatives by Cleveland Clinic and Partners Healthcare system.^31,32^ It identifies patients with CKD, phenotypically characterizes them, stores the information on laboratory tests and medications, calculates the validated Kidney Failure Risk Equation (KFRE)^33^ score, and updates automatically. The registry currently has 157,000 patients and provides the framework for identifying and addressing gaps in CKD care.

Our CKD PHM dashboard (Fig S1) includes population-based reports for CKD registry patients, with tabular displays of key metrics (eg, eGFR and KFRE score) and actionable tools to communicate with the clinical team. The dashboard allows users to sort patients and use filters such as primary care physician (PCP) group and risk status. The display of dates of last and next scheduled outreach allows active monitoring of follow-up status.

We created provider-facing dashboards for PCPs and nephrologists to facilitate the monitoring of clinicians’ patient panels (Fig S2). These dashboards can be run in real-time to track guideline-based metrics such as CKD stage, management (eg, albuminuria testing, blood pressure control), medication use, and vaccinations. Direct actionable links from the dashboard allow users to see review charts and facilitate addressing care gaps.

### Leveraging EHR to Facilitate CKD Recognition and Management

EHRs are integral for delivery and evaluation of PHM.^34^ A prior randomized controlled trial (RCT) conducted at UPMC tested clinical decision supports to improve care for patients with CKD stage 3b and 4 in the primary care setting. This intervention failed to increase albuminuria testing or nephrology referral.^35^ Subsequently, we implemented CKD care pathways and order sets in EHR to improve CKD recognition and management, but their clinical utilization was limited. The failure of these EHR tools to improve clinical care is perhaps not surprising because these are reported to cause alert fatigue and physician burnout and thus are often underutilized and ineffective.^36^ These experiences led us to design a novel PHM program for CKD *co-management* with primary care using the PHM informatics tools we have described.

The Kidney Coordinated Health Management Partnership (K-CHAMP) intervention included a remotely delivered, multifaceted bundle (conducted every 6 months) with (1) timely nephrology guidance and evidence-based recommendations through targeted automated e-consults (TACOs)^37,38^; (2) pharmacist-led medication management to improve safety, efficacy, ease of use, and affordability; and (3) nurse-led personalized patient education; as well as regular academic detailing for PCPs on advances in CKD management. The design strengths of the K-CHAMP approach have been previously reported and are briefly described in Box 2.^39,40^ This provider-centric approach was developed by incorporating feedback from PCPs and health system and health informatics leadership teams to ensure harmonization with PCP workflow. The e-consults provide recommendations to PCPs, who have the ultimate decision to place orders as deemed appropriate.

We tested the effectiveness of K-CHAMP intervention in a pragmatic, cluster RCT. Using an opt-out approach, we successfully enrolled 1,596 patients with CKD who were at high risk of progression to ESKD and were not seeing a nephrologist. The intervention improved the quality of CKD care by increasing prescriptions of kidney-protective medications, including angiotensin-converting enzyme (ACE) inhibitors/angiotensin receptor blockers (ARB) and SGLT2 inhibtors/GLP1 receptor agonists, but it did not reduce CKD progression, likely due to factors related to the COVID-19 pandemic and the short follow-up duration of 17 months.^39,41^ Our qualitative work among patients and PCPs showed high acceptance of the K-CHAMP intervention, support for collaborative CKD comanagement, and added value of timely access to specialty services and the multidisciplinary team, and it helped identify areas for future enhancements.

Recently, a large pragmatic RCT, ICD-Pieces, enrolled more than 11,000 patients with the triad of CKD, hypertension, and diabetes and showed that primary care practice facilitators improved some care delivery processes, including new ACE inhibitor/ARB use, but failed to improve the hospitalization rate at 1 year.^42^ Despite the negative results of both these large RCTs, they demonstrate a successful and feasible EHR-based PHM implementation model for conducting RCTs across a large number of primary care practices among highly complex patients.

### Translating Research Into Clinical Practice, Learning Health System Approach: UPMC CKD PHM Program

Recognizing the need for health-system-wide initiatives to improve CKD patient outcomes, care quality, and costs in UPMC’s integrated health system, we launched the Kidney-Care (K-Care) PHM program in 2021, with funding support from the UPMC Health Insurance Plan and approval from the quality improvement committee and PCP leadership. This program built on successful elements of K-CHAMP and used a learning health system approach to improve on its limitations. We enhanced the program by intensifying PCP education on the new CKD guidelines, individualizing CKD education for patients, adding a renal dietitian for CKD-specific diet education, adding a social worker to address social needs, and including referral to renal palliative care clinicians as needed.

As of June 2024, the K-Care program has enrolled approximately 3,000 patients (+70-80 new patients/month) from 101 PCP practices across 13 counties in western Pennsylvania, which includes about 25% rural patients. To manage the growth of the program in a cost-effective manner, we brought on a group of advanced practice providers (APPs) to do e-consults under the supervision of our nephrologists. Additionally, we integrated systematic screening and EHR documentation of social needs (medication assistance, financial assistance, transportation needs, and food insecurity) during each education encounter, with a trigger for renal social worker referral as appropriate. This program has high intervention fidelity and has performed over 6,600 e-consults, 5,600 medication reviews, and 4,700 education sessions over a 3-year period. Future work will evaluate the long-term clinical and cost effectiveness of this program.

## PHM Strategy for Kidney Palliative Care and Dialysis-Decision Making

Patients with advanced CKD have high palliative care needs, which often are not identified or addressed in part due to a shortage of palliative specialists. The burden instead falls on PCPs and nephrologists, who often have inadequate training, limited time, and competing demands.^43^ As a result, patients report high symptom burden, inadequate support in choosing treatment options for ESKD, and limited knowledge of alternative options such as medical management without dialysis.^44^ When many patients initiate dialysis, it does not align with their values and goals.^45^ Our prior qualitative work identified the need to build collaborative models of care and systematic triggers to identify the patients who may be appropriate for palliative care referral.^46^

In the K-Care program, we developed a systematic approach of identifying and initiating referrals to renal palliative care clinicians, who are APPs trained and supervised by dual–board-certified renal palliative physicians. Patients were triaged to a treatment decision making education session based on age (>80 years), CKD stage 4-5, and the surprise question (“Would you be surprised if the patient died in the next 12 months?”). The surprise question, a prognostication tool for mortality and hospitalization among patients with advanced CKD,^47^ was assessed by nephrology clinicians doing e-consults based on chart review, although it has not been explicitly validated for e-consultation. Our goal was to provide access to renal palliative care clinicians so that patients had an opportunity to have an informed shared decision-making discussion regarding ESKD treatment options that align with their values and preferences and to receive support for symptom management as appropriate. This unique model of care, which integrates renal palliative care in primary care for CKD patients, provides scalable access to these specialized services.

In our CKD clinics, we have implemented a similar process for automated referral to renal palliative care clinicians. This builds on our prior work of testing a clinical decision support with surprise question, which was successfully operationalized in the EHR but needed additional implementation efforts.^48^ Future work will evaluate the clinical and cost outcomes of these programs.

## PHM Implementation Strategy for Kidney Transplantation

Kidney transplantation (KT) remains the treatment of choice for patients with ESKD, and increasing access to KT is a key goal of the Advancing American Kidney Health initiative.^23^ We are developing a PHM-based approach to automate transplant referrals in our CKD clinic for all patients younger than 80 years with eGFR < 22 mL/min/1.73 m^2^. Nephrologists will be able to opt out if the patients are uninterested, have acute kidney injury, or have a life expectancy of less than 1 year. The goal of automated referrals is to ensure that all patients are given equal opportunity and timely access to mitigate any disparities and delays in KT referral.

Another potential area where PHM can be leveraged to improve KT outcomes is in addressing immunosuppression nonadherence, which is the main contributor to allograft failure.^49–51^ Several screening methods for nonadherence have been evaluated, including self-report, electronic pill monitoring, drug-level monitoring, pharmacy refill data, and missed laboratory testing,^52,53^ but there has been limited incorporation of these into routine clinical care; most transplant centers lack protocols for immunosuppression nonadherence screening and management.^54,55^ An EHR-based PHM approach can automate and standardize screening for nonadherence, integrate EHR tools for nonadherence risk stratification, and facilitate implementation of care pathways to address barriers to adherence. Such an approach aligns with the American Society of Transplantation Psychosocial Community of Practice Adherence Task Force recommendation for a multimodal approach.^53^

We are in the process of piloting programs for improving both KT referrals and outcomes.

## PHM Implementation Strategy for Optimal Transition to Kidney Replacement Therapy

Optimal transition to kidney replacement therapy (KRT) includes receiving a pre-emptive kidney transplant, initiating home dialysis (peritoneal dialysis or home hemodialysis), or initiating outpatient in-center hemodialysis via an arteriovenous fistula or graft. An optimal start is associated with better patient outcomes and lower health care costs.^56,57^ Unfortunately, in the United States, 85% of ESKD patients initiate hemodialysis with a catheter, and only about 15% do home dialysis or receive pre-emptive KT. Similarly, KT patients with a failing allograft have high rates of unplanned dialysis starts, and more than two-thirds initiate dialysis with a catheter.^58^

To optimize KRT transition, we plan to use EHR PHM to identify all eligible patients in our CKD clinic and use a multidisciplinary team model to ensure informed patient discussion of treatment options, timely referral for vascular or peritoneal dialysis access and transplant, care coordination, and identification and management of social risk factors.

## PHM Challenges and Future Directions

PHM can be an impactful strategy to improve value-based kidney care, but there are several financial and operational challenges in deploying this model of care in a fee-for-service environment.^25,59^ Successful implementation requires a strong infrastructure to support extensive EHR builds, data analytics, and clinical services and a strong commitment and upfront investment from health system leadership. This is a huge challenge in today’s environment, where many health systems are struggling financially in light of post–COVID-19 pandemic inflation and staffing shortages. The inherent VBC focus of PHM is unlikely to succeed unless there are policy changes that incentivize and promote VBC for upstream CKD care, with buy-in from key stakeholders (payors, physicians, and health systems). This is especially true as the overall cost of care with VBC is likely to increase due to newer high-cost medications; gains from averted cardiovascular complications or delaying progression to ESKD will occur years later.^60^ Moreover, besides costs, valid and meaningful quality metrics for evaluation of successful CKD care delivery and quality in VBCs are critically needed.^61^

To avoid physician burnout, a PHM approach that aligns with physician workflow and integrates behavior economics principles, such as report cards for PCPs and nephrologists and quality incentives based on achieving CKD care metrics, can help increase clinician engagement. Similarly, future work on incorporating robust methods for patient engagement and activation and using remote digital monitoring, mobile apps, and patient EHR portals is needed.

**Figure 3.**
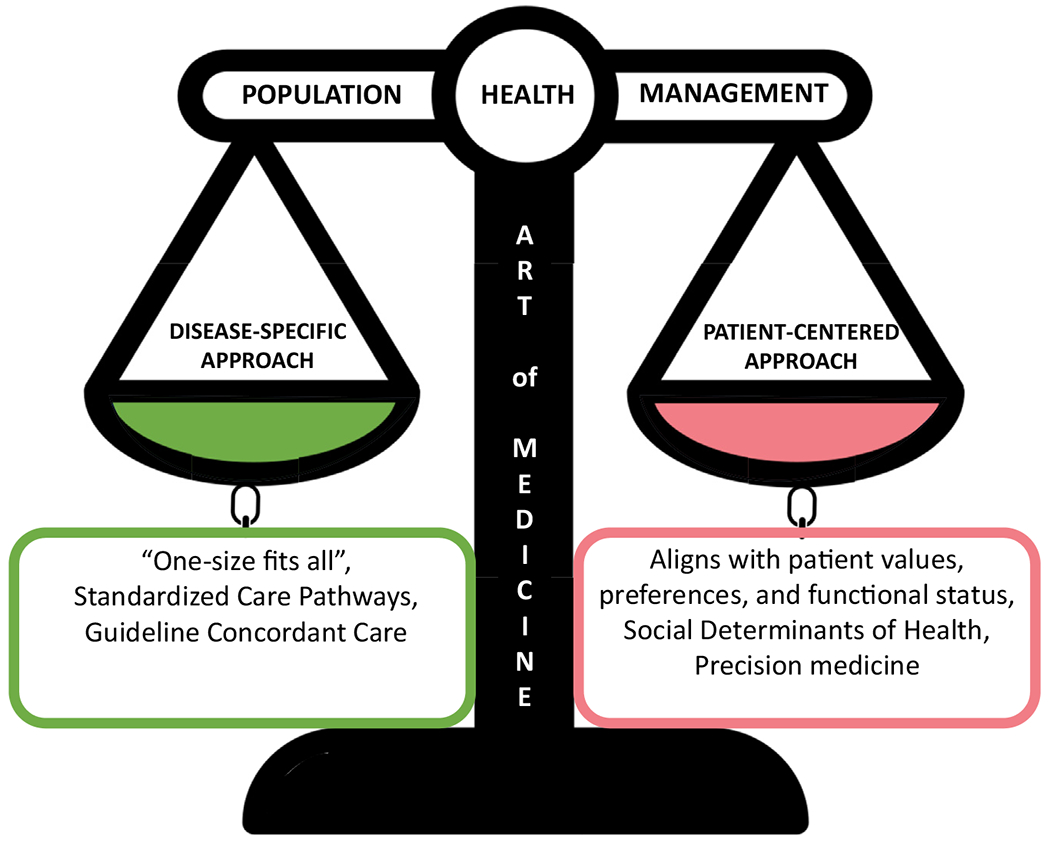
The promise of population health management approach to deliver patient-centered care. A holistic and equitable delivery of kidney health care can exist when population health management upholds the fine balance of disease-specific and patient-centered approaches centered by the “art of medicine.”

A potential pitfall of PHM is a one-size-fits-all approach focused on population and health-system outcomes rather than patient-centered outcomes. Although implementation of standardized care pathways may be desirable in most patients, careful consideration should be given to allow for individualizing treatment (Fig 3). This is integral to preserve the “art of medicine” and allow flexibility to tailor plans that align with patient values and preferences, which accounts not only for their disease state but also their functional status and social risk factors. Moreover, moving away from a disease-focused PHM to a person-centric holistic approach that targets cardio-kidney-metabolic syndrome is needed to optimize care and reduce care fragmentation. Future implementation research is needed to test PHM models of care that integrate multimorbidity disease care with patient-centered care and allow for opportunities for shared decision making.

Combining individualized plans of care with data-driven enhancements to PHM can help us move toward personalized medicine. Incorporating enhanced predictive modeling of risk using biomarker and genetic data, refining EHR phenotypes by incorporating natural language processing, and using machine-learning-based prediction models can help us better identify populations at risk. Identifying and addressing social determinants of health as part of clinical pathways can help mitigate health disparities in kidney disease and provide equitable access to specialized care. Prediction modeling of therapeutic response can help deliver precision medicine and help physicians choose the most effective drug for each patient, thus potentially optimizing medication effectiveness and safety and decreasing use of inappropriate medications.

## Conclusion

A thoughtfully designed PHM approach can potentially provide timely, equitable, resource- and cost-efficient care across health systems in a way that is scalable and data driven. Future work is needed to evaluate its short- and long-term clinical and cost-effectiveness and its impact on health disparities. Our management approach across the continuum of kidney disease illustrates how PHM can be a successful implementation strategy that modernizes care delivery. Future work should focus on addressing financial and operational challenges as well as enhancing patient and provider engagement.

## Supplementary Material

1 **Figure S1:** CKD PHM dashboard with integrated kidney failure risk equation.
**Figure S2:** CKD provider dashboard.

## Box 1. “Toolbox” With Critical Elements That Are Required to Establish a CKD PHM Program in a Health System

- Align goals of PHM program with health system priorities.
- Identify and empower clinical champions who can be instrumental “agents of change” and raise awareness of importance of establishing CKD as a priority.
- Identify and engage key stakeholders including health system, primary care, and nephrology leadership.
- Obtain financial support from the health system.
- Develop and implement standardized workflows with key input from clinical informatics, providers, and patients.
- Obtain necessary regulatory, compliance, and patient privacy approvals.
- Develop EHR infrastructure to identify care gaps, monitor program fidelity, and evaluate outcomes.
- Develop a strong clinical infrastructure with trained multidisciplinary team.
- Create standard management algorithms and update them frequently to incorporate latest evidence-based guidelines.

Abbreviations: CKD, chronic kidney disease; EHR, electronic health record; PHM, population health management.

## Box 2. Strengths of Our PHM Approach for CKD Management

- Standardizes management across a health system.
- Addresses multilevel barriers and provides resources at patient, provider, and health system levels.
- Optimizes population-level value-based CKD care based on latest evidence-based guidelines.
- Improves risk stratification and identification of care gaps and delivers resources to those at highest risk.
- Creates equitable access to specialized care and addresses disparities in care.
- Models a scalable care strategy over a large health system.
- Increases resource efficiency by creating a central highly skilled team.
- Leverages telemedicine to provide specialist access and education in patient’s existing primary care environment, thus minimizing care fragmentation and patient burden for travel.
- Provides individualized, patient-centered management plan through e-consults that account for factors such as insurance coverage, patient preferences, or functional status.
- Applies an opt-out approach to enroll PCPs and patients to minimize nonparticipation in the program.
- Uses automatically triggered e-consults (TACOs) to minimize PCP burden for nephrology referral.

Abbreviations: CKD, chronic kidney disease; PCP, primary care physician; PHM, population health management; TACOs, targeted automated e-consults.
