# Supplementary material for: Population Health Management for Improving Kidney Health Outcomes
Source: Am J Kidney Dis. Author manuscript; Available in PMC 2026 Jun 7. (PMC13242622; doi:10.1053/j.ajkd.2025.01.020)
Supplement: 1 — Figure S1: CKD PHM dashboard with integrated kidney failure risk equation. Figure S2: CKD provider dashboard. [file NIHMS2172384-supplement-1.pdf]

Figure S1. Chronic Kidney Disease Population Health Management dashboard with integrated Kidney Failure Risk Equation

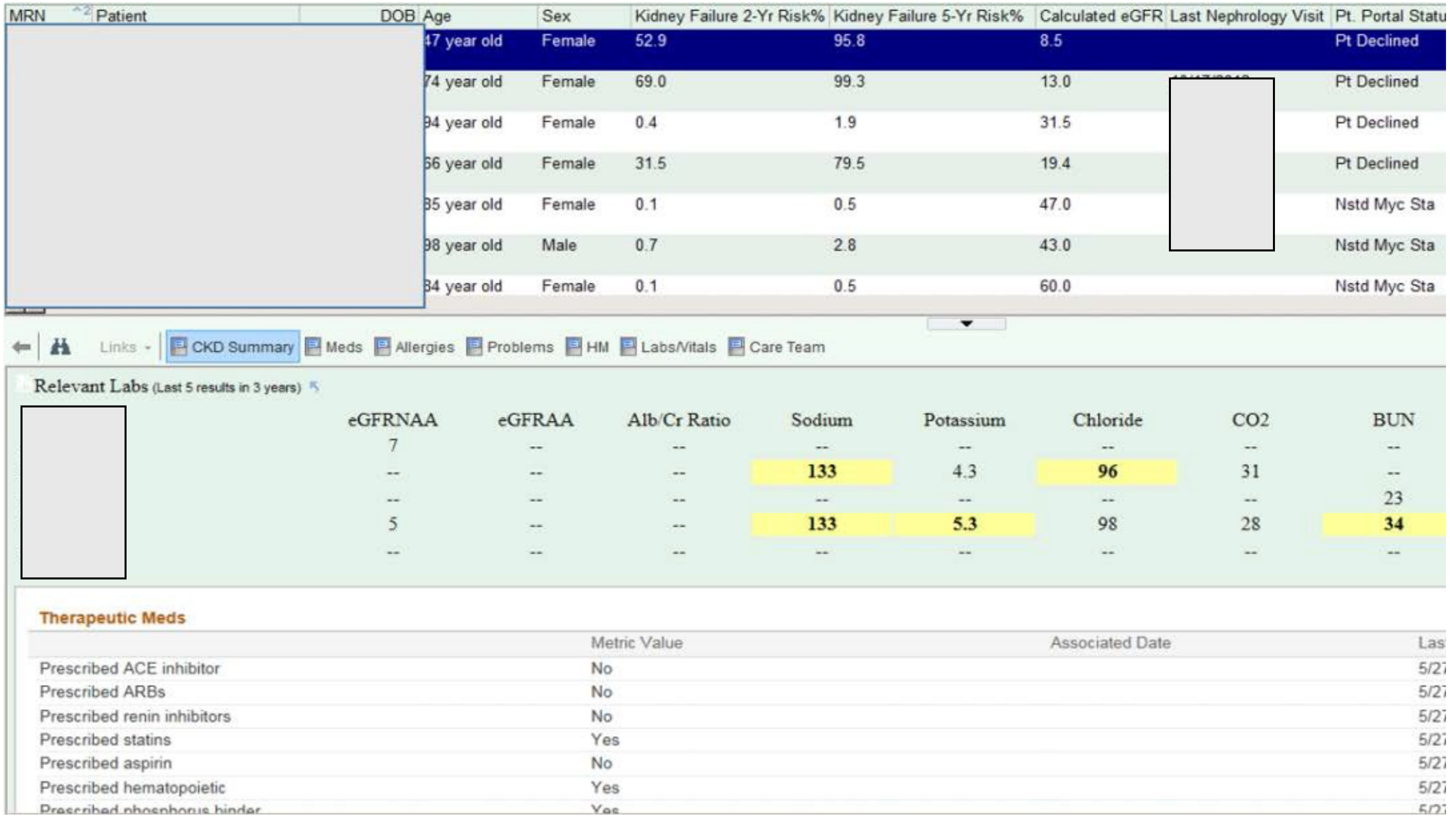

© 2023 UPMC*

**This material contains confidential and copyrighted information of Epic Systems Corporation (© 2023 Epic Systems Corporation).*

Figure S2. CKD provider Dashboard

Chronic Kidney Disease - Specialist

CKD Pts eGFR<15 w/Stage 5 CKD or ESRD

Last Refresh: 11:47:11 AM

Report completed: Mon 10/23 11:47 AM

CKD Stage 5 or ESRD	Total Pts	% of Total
CKD Stage 5 or ESRD	16	94.12 %
Not Coded CKD Stage 5 or ESRD	1	5.88 %

CKD Pts eGFR 15-29 w/Stage 4 CKD

Last Refresh: 11:48:18 AM

Report completed: Mon 10/23 11:48 AM

CKD Stage 4	Total Pts	% of Total
CKD Stage 4	43	87.76 %
Not Coded CKD Stage 4	6	12.24 %

CKD Pts eGFR Range 30-59 w/Stage 3 CKD

Last Refresh: 11:49:12 AM

Report completed: Mon 10/23 11:49 AM

CKD Stage 3	Total Pts	% of Total
CKD Stage 3	325	97.60 %
Not Coded CKD Stage 3	8	2.40 %

CKD Pts Last UACR

Last Refresh: 11:50:15 AM

Report completed: Mon 10/23 11:50 AM

Last eGFR NAA	Total Pts	% of Total
CKD Stage 3 eGFR Range 30-59	270	77.81 %
UACR 0-29	156	57.78 %
UACR 30-299	82	30.37 %
UACR 300+	32	11.85 %
CKD Stage 4 eGFR 15-29	38	10.95 %
UACR 0-29	8	21.05 %
UACR 30-299	16	42.11 %
UACR 300+	14	36.84 %

CKD Pts Last Hemoglobin

Last Refresh: 11:48:11 AM

Report completed: Mon 10/23 11:48 AM

Last Hemoglobin	Total Pts	% of Total
Hb <10	40	8.91 %
Hb >=10	393	87.53 %

CKD Pts UACR >30 On SGLT2i

Last Refresh: 11:48:36 AM

Report completed: Mon 10/23 11:48 AM

Last Urine Albumin:Creat Ratio	Total Pts	% of Total
UACR 30-299	111	67.27 %
Yes - Prescribed SGLT2i	26	23.42 %
No - Not Prescribed SGLT2i	85	76.58 %
UACR 300+	54	32.73 %
Yes - Prescribed SGLT2i	15	27.78 %
No - Not Prescribed SGLT2i	39	72.22 %

CKD Pts UACR >30 On Ace/Arb

Last Refresh: 11:49:03 AM

Report completed: Mon 10/23 11:49 AM

Last Urine Albumin:Creat Ratio	Total Pts	% of Total
UACR 30-299	88	67.18 %
No - Not Prescribed Ace/Arb	28	31.82 %
Yes - Prescribed Ace/Arb	60	68.18 %
UACR 300+	43	32.82 %
No - Not Prescribed Ace/Arb	17	39.53 %
Yes - Prescribed Ace/Arb	26	60.47 %

CKD Pts Age 60-85 on Low-Moderate-High Dose Statin

Last Refresh: 11:51:13 AM

Report completed: Mon 10/23 11:51 AM

On Low/ Moderate/ High Dose Statin	Total Pts	% of Total

CKD Pts Last Systolic/Diastolic BP

Last Refresh: 11:48:12 AM

Report completed: Mon 10/23 11:48 AM

Age	Total Pts	% of Total
Age <= 84	344	76.61 %
<130/80	135	39.24 %
130/80-140/90	117	34.01 %
>=140/90	79	22.97 %
No BP in Past Yr	13	3.78 %
Age 85+	105	23.39 %
<130/80	44	41.90 %
130/80-140/90	32	30.48 %
>=140/90	29	27.62 %
No BP in Past Yr	0	—

CKD Pts Flu Vaccine

Last Refresh: 11:48:49 AM

Report completed: Mon 10/23 11:48 AM

Overdue for Flu Vacc?	Total Pts	% of Total
Up to Date	132	29.80 %
Overdue	311	70.20 %

CKD Pts Pneumococcal Vaccine

Last Refresh: 11:49:43 AM

Report completed: Mon 10/23 11:49 AM

Pneumo overdue	Total Pts	% of Total
Up to Date	315	71.59 %
Overdue	125	28.41 %

CKD Total Population Report

Report completed: Mon 10/23 11:48 AM

CKD Total Population by PCP	449

© 2023 UPMC*

**This material contains confidential and copyrighted information of Epic Systems Corporation (© 2023 Epic Systems Corporation).*
